# Rates of motorcycle helmet use and reasons for non-use among adults and children in Luang Prabang, Lao People’s Democratic Republic

**DOI:** 10.1186/s12889-015-2304-2

**Published:** 2015-09-28

**Authors:** Michelle C. Fong, Jeffrey R. Measelle, Jessica L. Dwyer, Yvonne K. Taylor, Arian Mobasser, Theresa M. Strong, Susanne Werner, Siamphone Ouansavanh, Amphone Mounmingkham, Mai Kasuavang, Dalika Sittiphone, Khamhak Phoumesy, Keo Sysaythong, Khauphan Khantysavath, Somchit Bounnaphone, Amphone Vilaysom, Sengchanh Touvachao, Siviengxam Mounmeuangxam, Somchittana Souralay, Baoher Lianosay, Thongher Lia, Jonathan M. Spector

**Affiliations:** Lao Friends Hospital for Children, Luang Prabang, Lao, People’s Democratic Republic; Department of Psychology, University of Oregon, Eugene, OR USA; Department of General Pediatrics, Boston Children’s Hospital, Boston, USA

**Keywords:** Helmet use, Children, Motorcycles, Lao PDR

## Abstract

**Background:**

Motorcycles make up 81 % of the total vehicle population and 74 % of road traffic deaths in Lao PDR. Helmets reduce the risk and severity of injuries resulting from motorcycle accidents by 72 %. Although Lao law mandates motorcycle helmet use among drivers and passengers, the prevalence of helmet use in Luang Prabang, Lao PDR is unknown. This project aimed to measure the prevalence of motorcycle helmet use among riders (i.e., drivers and passengers) in Luang Prabang.

**Methods:**

An observational survey in Luang Prabang was conducted in February 2015 to measure the prevalence of motorcycle helmet use among drivers and passengers. Additionally, non-helmet wearing riders were surveyed to identify the reasons for helmet non-use.

**Results:**

Of 1632 motorcycle riders observed, only 16.2 % wore helmets. Approximately 29 % of adults wore helmets while less than 1 % of all children wore helmets. When surveyed about attitudes towards helmet use, the majority of adult drivers indicated that they did not like how adult helmets feel or made them look. Additionally, almost half of motorcyclists who did not own child helmets reported that their child was too young to wear a helmet.

**Conclusions:**

Our finding that children wear helmets at significantly lower rates compared to adults is consistent with findings from neighboring countries in Southeast Asia. Results of this study have implications for public health campaigns targeting helmet use, especially among children.

## Background

Road traffic injuries are a major public health problem and a leading cause of death around the world [1]. In Lao PDR, motorcycles are a common and integral means of transportation, making up 81 % of the total vehicle population [2]. As a result of the rapid growth in motorcycle use, there are increases in fatalities and injuries, particularly head injuries, among motorcyclists. Motorcyclists make up approximately 84 % of the total injured road users [3] and 74 % of road traffic deaths in Lao PDR [2]. Across Southeast Asia, mortality from road traffic injuries is estimated to be 7.4 deaths per 100,000 children [1].

**Fig. 1:**
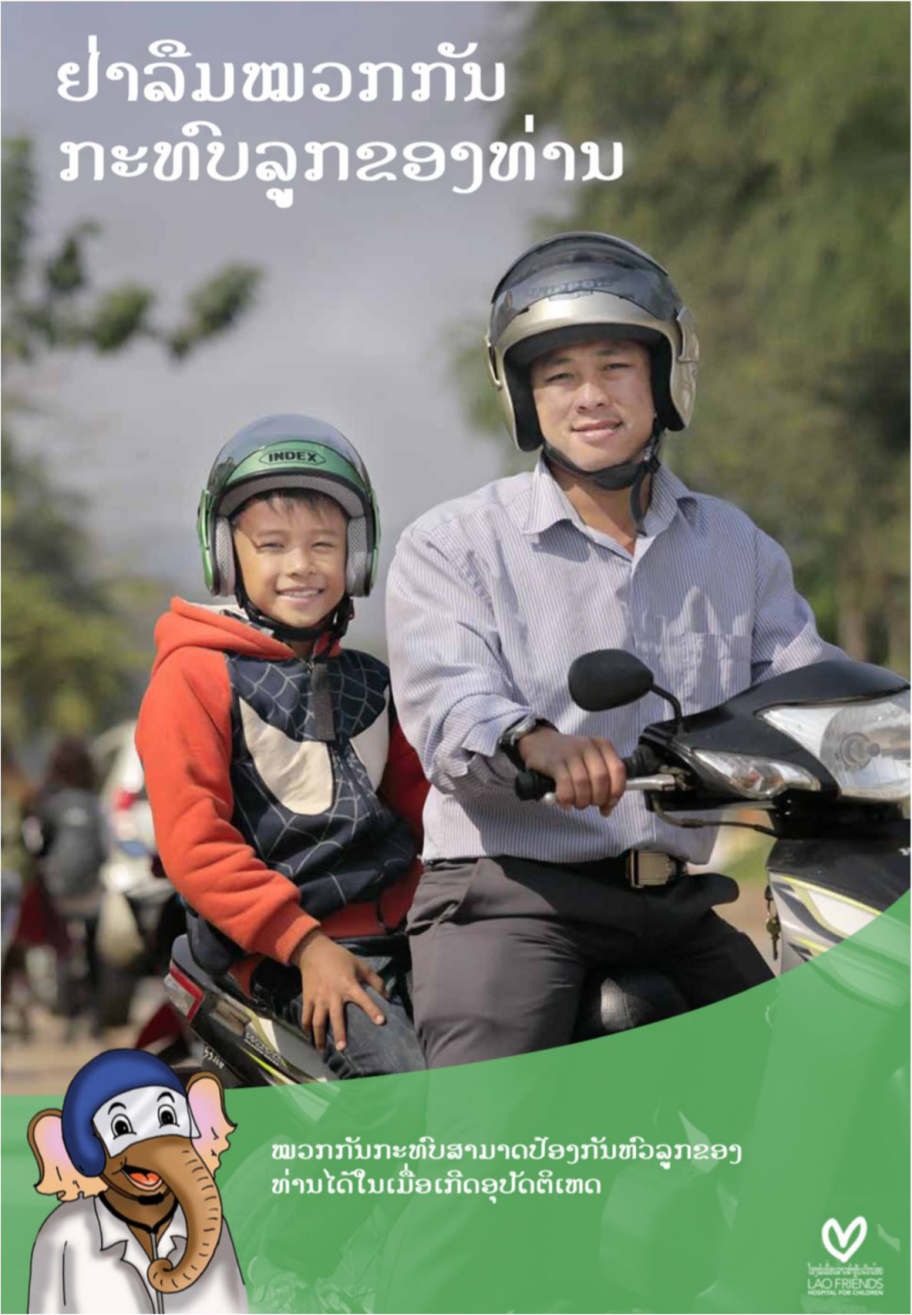
Lao educational poster to promote helmet use. This poster was developed at Lao Friends Hospital for Children in response to the findings of the current study. The main caption reads, “Remember your child’s motorbike helmet,” and the sub-caption reads, “A helmet will protect your child’s head in the event of an accident.” The elephant in the lower left hand corner of the poster, depicted wearing a helmet, is the hospital’s mascot

Injuries to the head and neck are the main cause of severe injury, disability or death among motorcyclists involved in road accidents [1]; approximately 88 % of motorcycle crash fatalities are due to head trauma [4]. Proper usage of motorcycle helmets is the single most effective way of preventing head injuries resulting from motorcycle accidents [1]. On motorcycles, helmets decrease the risk and severity of injuries by 72 %, decrease the likelihood of death by up to 39 % [1], and reduce the medical costs of injured riders and length of hospital stay [5].

Lao law allows a maximum of three riders per motorcycle and states that helmets are mandatory for all motorcycle riders, with a fine of 30,000 kip (about USD $4) for not wearing a “standard helmet while driving a motorcycle” [6, 7]. However, even with national legislation, the highest officially recorded helmet-wearing rate in the country’s capital of Vientiane was 76 % in 2008, though recorded rates have also ranged from 30 to 70 % [8]. Regional data on child helmet use rate presents a grimmer picture. In neighboring Vietnam, child helmet use rates were half the helmet use rates of adults [9]. No prior helmet studies have been conducted in Luang Prabang, Lao PDR, which has experienced a rapid increase in motorization in recent years.

This project aimed to measure the prevalence of motorcycle helmet use among riders (i.e., drivers and passengers) in the city of Luang Prabang, Lao PDR. Of particular interest was information about child helmet practices as it is common in Southeast Asia for motorcycles to serve as families’ primary mode of transportation. A second objective of the current study was to conduct post-observation surveys of non-helmet wearing riders to identify reasons for non-helmet use. The study was conducted by healthcare professionals at the Lao Friends Hospital for Children (LFHC), a new pediatric medical center in Luang Prabang that was built in partnership with the Lao PDR Ministry of Health. It was anticipated that results would help inform broad-based public health interventions that target efforts at greater awareness and behavior change among families seeking primary care services in the area.

## Methods

### Procedure and materials

Motorcycle helmet use and non-use data was collected by census teams in four locations near schools and on main streets in Luang Prabang, Lao PDR. To capture variations in motorcycle helmet use across the day, data were collected across a three-hour morning (7:00-10:00 am) and three-hour afternoon (3:00-6:00 pm) period on 06 February 2015. Driver and passenger age (adult or child), as well as helmet use and non-use information were recorded for drivers and passengers of every fifth motorcycle entering in four locations. Motorcycle operators were coded as drivers while all other motorcycle riders were coded as passengers [1]. Gender was not recorded, as it was not feasible for our census teams to record gender observations accurately in addition to our more immediate goal of capturing age estimates and helmet use information for all motorcycle drivers and passengers. Census teams comprised three to four healthcare professionals from LFHC who were positioned on opposite sides of the road to account for bidirectional traffic flow patterns. Each census team was assigned one location and attempted to collect helmet use data for at least 200 motorcycles. Observation sites were selected to be representative of traffic patterns, to increase generalizability of findings.

Additionally, 100 non-helmet wearing adult motorcyclists with child passengers were approached at three local markets and asked if they would be willing to be interviewed about motorcycle helmet ownership and use. Interview sites were selected next to local markets as they were areas where motorcycles slowed down or stopped, allowing research staff to approach motorcyclists. The helmet use survey was piloted in an earlier study and subsequently revised to include questions about adult and child motorcycle helmet ownership (e.g., “Do you own a child’s motorcycle helmet?”), and reasons for helmet non-use (e.g., “Why do you not own a child’s helmet?”). For questions about motorcycle helmet ownership, researchers marked “yes” or “no” according to motorcyclists’ response. Motorcyclists who reported owning a motorcycle helmet were asked why they did not always wear helmets while riding motorcycles. Similarly, motorcyclists who reported not owning a motorcycle helmet were asked why they did not own a motorcycle helmet. Motorcyclists also responded to the same set of questions with respect to child motorcycle helmet use. Researchers marked one or more options from a list of pre-defined answers (e.g., “do not like how it feels”), based on motorcyclists’ responses to the questions. Responses that did not fit into any pre-defined answers were marked as “other”.

In the present study, drivers and passengers who appeared to be under 15 years of age were categorized as children. Drivers and passengers who could not be clearly identified as less than 15 years of age were coded as adults (note: 15 years of age is considered the legal age to operate a motorcycle [6]). Information about driver or passenger gender was not collected.

Approval for this study was provided two ways. First, the National Ethics Committee through the Lao Ministry of Health approved health survey research with Lao citizens in support of the public health mission of LFHC (Protocol 002NIOPH/NECHR). Second, the University of Oregon Institutional Review Board and Research Compliance Services Office approved the Lao-based research activities of authors Fong and Measelle, who played a central role in the design and execution of the current study (UO Protocol 12012014.002). Following the World Health Organization’s research ethics review guidelines (http://www.who.int/rpc/research_ethics/erc/en/), roadside observations of helmet use in this study were observations of public behavior (i.e., travelling on a public road) in which the participants could not be identified. As such, consent was not obtained for roadside observations of helmet use. Oral informed consent was obtained from all participants in the helmet use surveys.

### Statistical analysis

SPSS version 20 (IBM Corp, Armonk, NY) was used to conduct all data analyses. Frequencies and proportions for helmet use by variables including age (adult or child) and rider type (driver or passenger) were calculated. Chi-square tests were used to examine the proportional difference between driver ages, passenger ages, and helmet use, where appropriate. A paired samples *t*-test was used to compare the mean number of child passengers relative to the number of adult passengers on passenger-carrying motorcycles.

## Results

### Roadside observations

We observed 846 motorcycles, with 846 drivers and 786 passengers, totaling 1632 riders. Table 1 provides the proportions of adult and child riders and distinguishes between driver and passenger, as well as riders who were and were not wearing helmets.

**Table 1:** Motorcycle helmet use frequency in Luang Prabang

	Adults *% (n)*			Children *% (n)*			Helmet use *% (n)*		
	Helmet used	Without helmet	Sub-total	Helmet used	Without helmet	Sub-total	Helmet used	Without helmet	Total
Drivers	27.5 (251)	58.5 (534)	86.0 (785)	0.3 (2)	8.2 (59)	8.5 (61)	15.5 (253)	36.3 (593)	51.8 (846)
Passengers	1.2 (11)	12.8 (117)	14.0 (128)	0.1 (1)	91.4 (657)	91.5 (658)	0.7 (12)	47.4 (774)	48.2 (786)
Sub-total	28.7 (262)	71.3 (651)	100 (913)	0.4 (3)	99.6 (716)	100 (719)	16.2 (265)	83.8 (1367)	100 (1632)

The number of riders per motorcycle ranged from 1 to 5, with 69.4 % of motorcycles carrying one passenger, 20.3 % of motorcycles carrying two passengers, 2.8 % of motorcycles carrying three passengers, and 0.4 % carrying four passengers. 67.8 % of adult drivers carried one or more passengers whereas and 90.2 % of child drivers carried one or more passengers. When carrying passengers, adults drivers tended to carry significantly more passengers (*M* = 1.36 passengers, *SD* = 0.80) compared to child drivers (*M* = 1.13 passengers, *SD* = 0.47; *t*(585) = 2.93, *p* = .004). Overall, there were significantly more child (*M* = 1.12, *SD* = 0.34) than adult (*M* = 0.22, *SD* = 0.56) passengers (*t*(586) = 22.93, *p* < .001).

### Helmet use

Out of all riders, only 16.2 % wore helmets (Table 1). 28.7 % of adults wore helmets and whereas 0.4 % of children wore helmets. The overall prevalence of drivers’ helmet use was 15.5 %. Significantly more adult drivers rode without helmets (58.5 %) than with helmets (27.5 %; *χ*^2^(1) = 102.02, *p* < .001). Similarly, significantly more child drivers rode without helmets (8.2 %) than with helmets (0.3 %; *χ*^2^(1) = 53.26, *p* < .001). The proportion of adult drivers wearing helmets (27.5 %) was significantly greater than the proportion of child drivers wearing helmets (0.3 %; *χ*^2^ (1) = 22.24, *p* < .001).

The prevalence of passengers’ helmet use was 0.7 %. Significantly more adult passengers rode without helmets (12.8 %) than with helmets (1.2 %; *χ*^2^(1) = 219.81, *p* < .001). Similarly, significantly child passengers more rode without helmets (91.4 %) than with helmets (0.1 %; *χ*^2^(1) = 481.93, *p* < .001). In all comparisons of passengers wearing helmets to passengers not wearing helmets, the proportion of passengers without helmets (47.4 %) was significantly greater than the proportion of passengers wearing helmets (0.7 %; *χ*^2^(1) = 242.28 to 522.69, *p*s < .001).

### Helmet use surveys

#### Attitudes towards adult helmet use

Following the observational sessions, 100 non-helmet wearing adult drivers were approached about participating in a motorcycle helmet survey. Of these, 91 (91 %) agreed to be surveyed about their helmet usage and attitudes toward helmets. Of these, 79 drivers (86.8 %) reported owning a motorcycle helmet whereas 12 drivers (13.2 %) reported that they did not own a helmet. When asked how often they wear a helmet (“never”, “sometimes”, “half of the time”, “most of the time” or “always”), drivers owning helmets typically reported that they only “sometimes” wear helmets (*M* = 1.33, *SD* = .94, range = 0-4). Significantly more drivers (54.8 %) indicated that they “never” or “sometimes” wear a helmet compared to drivers who indicated that they wear a helmet “half of the time” or more (45.2 %), *χ*^2^(1) = 9.98, *p* = .002.

Table 2 provides a breakdown of the reasons adult helmet owners offered for not wearing a helmet. Respondents were allowed to provide more than one reason for motorcycle helmet non-use. A significant majority (51.9 %) indicated that they did not like how a helmet feels or how it makes them look, compared to drivers who indicated that it interferes with their driving (29.1 %), *χ*^2^(1) = 21.08, *p* < .001. Approximately 6 % indicated that helmets were not necessary for safety reasons.

**Table 2:** Reasons for adult motorcycle helmet non-use

	Owns adult helmet	Does not own adult helmet
	*%* (*n*)	*%* (*n*)
Too expensive	-	4.5 (1)
Not necessary for safety reasons	5.8 (6)	9.1 (2)
Do not like how it feels	31.7 (33)	40.9 (9)
Interferes with my driving	29.8 (31)	13.6 (3)
Interferes with how I look	20.2 (21)	13.6 (3)
Will get stolen	2.9 (3)	4.5 (1)
Other	9.6 (10)	13.6 (3)

Of the 91 adult drivers surveyed, 79 reported owning an adult motorcycle helmet and 12 reported not owning an adult motorcycle helmet. Drivers could provide more than one reason for not owning a helmet

Table 2 also breaks down the reasons offered for not owning an adult helmet. Here too, a significant majority (54.5 %) indicated that they did not like how a helmet feels or how it makes them look compared to riders who indicated that it interfered with their driving (13.6 %), *χ*^2^(1) = 36.26, *p* = .009. Approximately 9 % non-owning riders indicated that helmets were not necessary for safety reasons.

### Attitudes towards child helmet use

The adult drivers being surveyed were asked if they owned a child motorcycle helmet; a total of 15 drivers (16.5 %) reported owning a child helmet. When asked how often their child passengers wore a helmet, 10 % said “never” while 90 % said “sometimes.”

Table 3 provides a breakdown of the reasons why child passengers were not wearing a helmet even if the surveyed driver indicated that a child helmet was owned. Although a range of explanations were offered, a significant number (63.7 %) indicated that their child did not wear a helmet because their child was either too young, their child refused to wear a helmet, or, more typically, for both reasons, *χ*^2^(2) = 13.00, *p* < .002.

**Table 3:** Reasons for child motorcycle helmet non-use

	Owns child helmet	Does not own child helmet
	*%* (*n*)	*%* (*n*)
Too expensive	-	9.9 (10)
Not necessary for safety reasons	4.5 (1)	8.9 (9)
Cannot find a child’s helmet	-	18.8 (19)
My child is too young	36.4 (8)	32.7 (33)
My child refuses to wear the helmet	27.3 (6)	18.8 (19)
No place to store the helmet	13.6 (3)	7.9 (8)
Other	18.2 (4)	3.0 (3)

Of the 91 adult drivers surveyed, 15 reported owning a child’s motorcycle helmet and 76 reported not owning a child’s motorcycle helmet. Drivers could provide more than one reason for not owning a child’s helmet

Table 3 also breaks down the reasons offered why riders did not own a child helmet. Most respondents provided more than one response, yet 32.7 % responded that their child was too young to wear a helmet; this response was given significantly more frequently than any other single response, *χ*^2^(2) = 93.61-143.99, *p*s < .001.

## Discussion

Although Lao law mandates that all motorcyclists must wear helmets [6], we found that a small minority of all motorcycle riders in Luang Prabang (16.2 %) wore helmets. In Vientiane, the capital of Lao PDR, estimates of helmet use rates vary according to the data source. For example, the government reported helmet use rates of 70 % [10] for the month of July 2005 in Vientiane, while a non-government organization recorded rates of 37 % in the same month [11]. When limiting our sample to adult drivers, we found helmet use rates of 32 %. However, when including all drivers and passengers, we found an overall helmet use rate of only 16.2 %.

We observed less than 1 % of all children wearing helmets. Our finding that children wear helmets at lower rates compared to adults is consistent with findings from neighboring Vietnam, where approximately 30 to 50 % of children wear motorcycle helmets and over 90 % of adults wear motorcycle helmets [9]. In the current study, the most common reasons reported by adults for children not wearing helmets were “my child is too young” and “my child refuses to wear the helmet”. Although a small percentage of respondents did not endorse the safety value of helmets for children, the combined set of reasons provided for why children were not wearing helmets contributed to an alarmingly high number of helmet-less children. These findings may help to inform future interventions to increase rates of helmet use. Specifically, interventions may benefit from conveying the importance of helmets for children [1], emphasizing that even young children should wear helmets. Additionally, given that two-thirds of adults in the sample did not wear helmets, interventions may need to stress the importance of adults as helmet-wearing role models for children. Based on these results, efforts are underway to develop public health messages aimed at patients seeking care at LFHC. Educational posters to promote helmet use were newly designed and now hang prominently in clinical care areas (see Fig. 1).

Another important finding of the study was that 29 % of motorcyclists who did not own child helmets reported that child helmets were too expensive or difficult to find. That access to child helmets was mentioned repeatedly as a barrier to use suggests that public and private efforts to increase both the availability and access to child helmets (e.g., donation campaigns and/or the provision of helmets at low cost) could improve helmet use among children.

Finally, we observed 61 child drivers (7.2 % of all drivers), which is concerning given that 15 years is the legal minimum age for motorcycle operation. Only two of the child drivers observed used a helmet. This is likely to be a conservative estimate of child drivers given that drivers and passengers who could not be clearly identified as less than 15 years of age were coded as adults. Also of concern, we observed 24 motorcycles (2.8 %) with four riders and 3 motorcycles (0.4 %) with five riders, despite Lao law that allows a maximum of three riders per motorcycle. In general, these figures suggest that the laws regulating motorcycle ridership and helmet usage are either poorly understand or generally discounted given a combination of reasons, including attitudes and beliefs (e.g., interferences with looks; not liking how helmets feel) as well as practical constraints (e.g., availability).

Limitations of the current study include use of cross-sectional data collected in the course of a single day. While the study was designed to capture variations in helmet use that may occur throughout the day, we could not account for seasonal differences. The present data were collected during the dry season; increases or decreases in helmet use may occur in the rainy season. Another limitation is the focus on an urban sample of motorcyclists. Luang Prabang was chosen for observation because of the increased prevalence of motorcyclists compared to rural areas. It is unknown whether rates of helmet use in rural parts of the country would differ, though we suspect that access to helmets in rural settings may be lower compared to urban settings. Additionally, prior research in middle-income countries such as Malaysia [12] and China [13] have found riders to wear helmets at higher rates in urban vs. rural settings. Finally, we did not gather information to interpret endorsements of the response option “my child is too young [to wear a helmet]”. It is possible that parents think that a helmet could be too heavy and may hurt a developing child’s neck. For example, in Vietnam, there are beliefs that helmets increase the risk of neck injury in children [14]. As such, parents who endorse the statement may also be concerned about safety. However, we are unable to make this interpretation without additional data.

## Conclusions

In summary, our observations suggest the prevalence of motorcycle helmet use among all riders, and among children in particular, might be very low in Luang Prabang despite mandatory laws regarding motorcycle helmet use. We have started to develop educational materials to promote helmet use. Next steps may include broader educational programs and/or helmet distribution campaigns aimed at increasing helmet use among adults and children.
